# Editor-in-Chief’s Letter to Readers and Authors of *Marine Drugs*

**DOI:** 10.3390/md19080422

**Published:** 2021-07-26

**Authors:** Orazio Taglialatela-Scafati

**Affiliations:** Department of Pharmacy, School of Medicine and Surgery, University of Naples Federico II, Via Montesano 49, 80131 Naples, Italy; scatagli@unina.it

In recent weeks, Scopus and Journal Citation Reports (JCR)/Science Edition (Clarivate Analytics) have released the bibliometric parameters CiteScore and Impact Factor, respectively. In both cases, *Marine Drugs* [1] has experienced a remarkable score increase (CiteScore from 5.1 to 6.4; Impact Factor from 4.073 to 5.118). *Marine Drugs* is now Q1 in the three Scopus categories “Pharmacology, Toxicology and Pharmaceutics”, “Pharmaceutical Science”, and “Drug Discovery”. *Marine Drugs* is also Q1 in the WoS JCR category “Chemistry, Medicinal” and in the newly reached category “Pharmacology & Pharmacy”.

These great outcomes are certainly due to the high quality of experimental manuscripts and reviews that our Authors submit to the journal and to the invaluable help and support from the Editorial Board Members, coordinated through the careful scrutiny of the Associate Editors (Prof. Dr. Bill J. Baker, Prof. Dr. Marc Diederich, Prof. Dr. Anake Kijjoa, Dr. Ipek Kurtboke, Prof. Dr. Marialuisa Menna, Prof. Dr. Vassilios Roussis, Dr. Hitoshi Sashiwa, and Prof. Dr. Claudiu T. Supuran). However, the process and performance of the journal are dependent upon the activity of the Editorial Office, an efficient team working hard to maintain the relationships with Authors, Reviewers and Editors. These are crucial factors for obtaining results such as a median time from submission to first decision of 11.7 days (first half of 2021). 

The highest Impact Factor of the journal history comes almost 20 years after its launch (*Marine Drugs* is one of the “oldest” MDPI journals). The inaugural issue of *Marine Drugs* was released in November 2003 and the first article [2], entitled “*Marine Drugs*—A New International Journal”, was authored by Dr. Shu-Kun Lin and Dr. Derek J. McPhee. Prof. Dr. Hua-Shi Guan of the Ocean University of China, Qingdao, was the founding Editor-in-Chief and directed the journal for 2 years, obtaining coverage by Science Citation Index Expanded (SCIE) and, later, by PubMed. Two German scholars led the journal from 2005 to 2013 (Prof. Dr. Peter Proksch from 2005 to 2009, and Prof. Dr. Hartmut Laatsch from 2009 to 2013), profoundly shaping the journal profile, recruiting prestigious Associate Editors, and receiving high impact factors (3.978 in 2013). In the following 4-year term (2014–2018), the journal was led by my predecessor Prof. Dr. Alejandro M. Mayer, to whom I am profoundly indebted for offering me the Associate Editor position. Under Mayer’s leadership, the journal gained further prestige and impact, sponsored important conferences, and collaborated with societies in the field. 

My efforts in recent years have been devoted to updating the journal with regard to recent trends in marine research, expanding its aims and scopes, and hosting several papers on new subject areas such as marine chemical ecology research to inspire marine drug discovery as well as the use of biomaterials of marine origin and marine-derived ingredients for cosmeceuticals, nutraceuticals, and nutritional supplements, etc.

It is now time to look into the future, identify further development strategies, and work to shape a journal that remains the reference forum for the publication of high-quality papers in the fascinating and rapidly changing area of marine-derived metabolites. *Marine Drugs* looks forward to your continuous input and contributions.
